# Probing the putative α7 nAChR/NMDAR complex in human and murine cortex and hippocampus: Different degrees of complex formation in healthy and Alzheimer brain tissue

**DOI:** 10.1371/journal.pone.0189513

**Published:** 2017-12-20

**Authors:** Mohamed R. Elnagar, Anne Byriel Walls, Gouda K. Helal, Farid M. Hamada, Morten Skøtt Thomsen, Anders A. Jensen

**Affiliations:** 1 Department of Drug Design and Pharmacology, Faculty of Health and Medical Sciences, University of Copenhagen, Universitetsparken, Copenhagen Ø, Denmark; 2 Faculty of Pharmacy, Al-Azhar University, Al-Mokhaym Al-Daem, Nasr City, Cairo, Egypt; Weizmann Institute of Science, ISRAEL

## Abstract

α7 nicotinic acetylcholine receptors (nAChRs) and *N*-methyl-D-aspartate receptors (NMDARs) are key mediators of central cholinergic and glutamatergic neurotransmission, respectively. In addition to numerous well-established functional interactions between α7 nAChRs and NMDARs, the two receptors have been proposed to form a multimeric complex, and in the present study we have investigated this putative α7 nAChR/NMDAR assembly in human and murine brain tissues. By α-bungarotoxin (BGT) affinity purification, α7 and NMDAR subunits were co-purified from human and murine cortical and hippocampal homogenates, substantiating the notion that the receptors are parts of a multimeric complex in the human and rodent brain. Interestingly, the ratios between GluN1 and α7 levels in BGT pull-downs from cortical homogenates from Alzheimer’s disease (AD) brains were significantly lower than those in pull-downs from non-AD controls, indicating a reduced degree of α7 nAChR/NMDAR complex formation in the diseased tissue. A similar difference in GluN1/α7 ratios was observed between pull-downs from cortical homogenates from adult 3xTg-AD and age-matched wild type (WT) mice, whereas the GluN1/α7 ratios determined in pull-downs from young 3xTg-AD and age-matched WT mice did not differ significantly. The observation that pretreatment with oligomeric amyloid-β_1–42_ reduced GluN1/α7 ratios in BGT pull-downs from human cortical homogenate in a concentration-dependent manner provided a plausible molecular mechanism for this observed reduction. In conclusion, while it will be important to further challenge the existence of the putative α7 nAChR/NMDAR complex in future studies applying other methodologies than biochemical assays and to investigate the functional implications of this complex for cholinergic and glutamatergic neurotransmission, this work supports the formation of the complex and presents new insights into its regulation in healthy and diseased brain tissue.

## Introduction

Glutamate (Glu) and acetylcholine (ACh) are major neurotransmitters in the central nervous system (CNS), where both are directly involved in or regulate a wide spectrum of physiological processes. Dysfunctions in glutamatergic and cholinergic neurotransmission have been implicated in numerous pathological states, and modulation of glutamatergic and/or cholinergic mechanisms holds considerable therapeutic potential when it comes to numerous cognitive, psychiatric and neurodegenerative disorders [1–4]. Both Glu and ACh mediate their physiological effects through highly heterogeneous families of G-protein coupled receptors (GPCRs) and ligand-gated cation channels (LGICs). Glu acts through three classes of LGICs: the *N*-methyl-*D*-aspartate (NMDA), α-amino-3-hydroxy-5-methyl-4-isoxazolepropionate (AMPA) and kainate receptors. The receptors are heteromeric complexes assembled by four subunits, with the prototypic NMDA receptor (NMDAR) being comprised of two GluN1 subunits and two GluN2 subunits (GluN2A-2D) [5]. The fast cholinergic signalling in the CNS is mediated by the neuronal nicotinic ACh receptors (nAChRs) that are homo- and heteropentameric complexes assembled from α2-α10 and β2-β4 subunits, with the homomeric α7 nAChR being one of the two major physiological neuronal nAChR subtypes [3, 4].

A substantial amount of experimental data has established NMDARs and α7 nAChRs as key contributors to the glutamatergic and cholinergic components of cognitive functions. NMDARs are the key mediators of the long-term potentiation (LTP) believed to be at the very core of synaptic plasticity and cognition [6], and a close link between α7 nAChRs and glutamatergic transmission exists due to functional interactions at numerous levels, with presynaptic α7 nAChRs facilitating Glu release throughout the brain and α7 nAChRs being capable of modulating synaptic plasticity through NMDARs [3, 7–10]. In addition to this functional cross-talk and synergy between the two receptors, Liu and colleagues have found NMDARs and α7 nAChRs to assemble into receptor complexes [11–13]. The GluN2A subunit has been shown to coimmunoprecipitate with the α7 subunit from rat hippocampal homogenate, and GluN2A and α7 have been proposed to form a direct protein-protein interaction rooted in the carboxy terminal of GluN2A and a segment of the second intracellular loop of α7 [11]. Based on studies applying a peptide (α7-pep2) that selectively interferes with the α7 nAChR/NMDAR assembly, the complex formation has been proposed to underlie α7 nAChR-mediated augmentation of NMDAR-mediated whole-cell currents and miniature excitatory postsynaptic currents of LTP in cultured hippocampal cultures [12]. Moreover, the α7 nAChR/NMDAR coupling has been proposed to be important for cue-induced nicotine reinstatement and to affect some cognitive domains [11, 12].

The putative complex formation between NMDARs and α7 nAChRs and the possibilities for direct cross-talk between the two receptors as well as between the glutamatergic and cholinergic systems arising from it present interesting perspectives. In the present study, we have investigated the assembly of the complex in murine and human cortical and hippocampal brain tissues in affinity purification experiments. The degrees of α7 nAChR/NMDAR complex formation in cortical homogenates from healthy control (non-Alzheimer’s Disease [non-AD]) and AD humans and in wild-type (WT) and 3xTg-AD mice brain cortical homogenates have been also compared, and the molecular mechanism underlying the observed differences in complex formation between these tissues has been investigated.

## Materials and methods

### Chemicals

Bovine serum albumin (BSA) and all chemicals used in the buffers were obtained from Sigma-Aldrich (Brøndby, Denmark). α-bungarotoxin (BGT) was obtained from Tocris Cookson (Bristol, UK). The Aβ_1–42_, α7-pep1 and α7-pep2 peptides were purchased from Dg-Peptide Co. (Hang Zhou City, China).

### Human brain tissue

**Fig 1:**
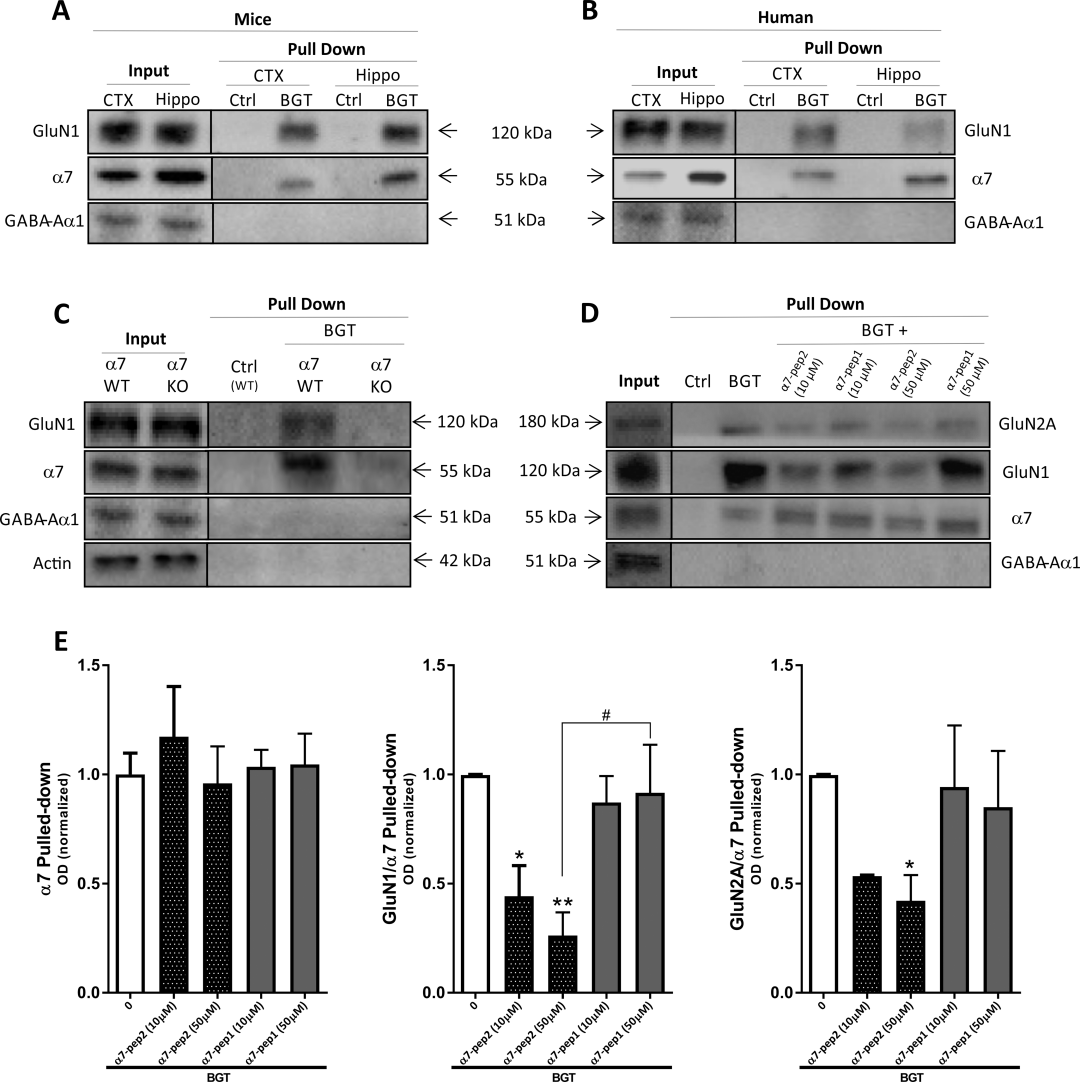
Complex formation between α7 nAChR and NMDAR in murine and human cortex and hippocampus. **(A-B)** Affinity purification with agarose beads covalently coupled with α-bungarotoxin (BGT) or BSA (Ctrl) on homogenates from murine (A) and human (B) cortical (CTX) and hippocampal (Hippo) tissues. Total lysates (Input) and pulled-down (Pull Down) samples were submitted to gel electrophoresis and Western blotting followed by detection using antibodies for GluN1, α7 nAChR and GABA_A_R α1 subunits. The gels in A and B are representative for different experiments using tissues from 4 different mouse hippocampi, 4 different mouse cortices, 2 different human hippocampi and 2 different human cortices. (**C**) Total lysates (Input) and pulled-down (Pull Down) samples from WT and α7 KO mouse cortical homogenates were submitted to gel electrophoresis and Western blotting followed by detection using antibodies for GluN1, α7 nAChR and GABA_A_R α1 subunits and β-actin. (**D**) Total lysates (Input) and pulled-down (Pull Down) samples from mouse cortical homogenates pretreated with buffer or buffer supplemented with α7-pep2 (10 μM and 50 μM) or α7-pep1 (10 μM and 50 μM) were submitted to gel electrophoresis and Western blotting followed by detection using antibodies for GluN1, GluN2A, α7 nAChR and GABA_A_R α1 subunits. (**E**) Quantification of α7 pulled-down and GluN1 and GluN2A pulled-down (normalized to the pulled-down α7) from mouse cortical homogenates pretreated with buffer or buffer supplemented with α7-pep2 (10 μM and 50 μM) or α7-pep1 (10 μM and 50 μM). Values are given as mean ± SEM (n = 3–4, i.e. 3–4 different mouse cortices, the experiment was performed once). *p <0.05 and **p < 0.01 indicate statistically significant difference from the vehicle-treated group in Kruskal-Wallis test with Dunn’s multiple comparison test. ^#^p <0.05 indicates statistically significant difference between GluN1/α7 Pulled-down ratios between α7-pep2 (50 μM) or α7-pep1 (50 μM) in unpaired *t*-tests.

Human temporal cortical and hippocampal tissues used for the experiments in Figs 1B and 5 were obtained by surgery from subjects with medically intractable temporal lobe epilepsy with hippocampal onset. The hippocampus tissues were obtained from two subjects (1 female aged 41 years and 1 male aged 54 years), and the cortical tissues were obtained from four subjects (3 females aged 41, 55 and 57 years and 1 male aged 54 years). Written informed consent was obtained from all patients before surgery. The study was approved by the Ethical Committee in the Capital Region of Denmark (H-2-2011-104) and performed in accordance with the Declaration of Helsinki. The tissues were dissected and immediately frozen on dry ice and stored at -80°C until use. The neuropathologic examinations of the neocortex from all patients were normal.

**Fig 2:**
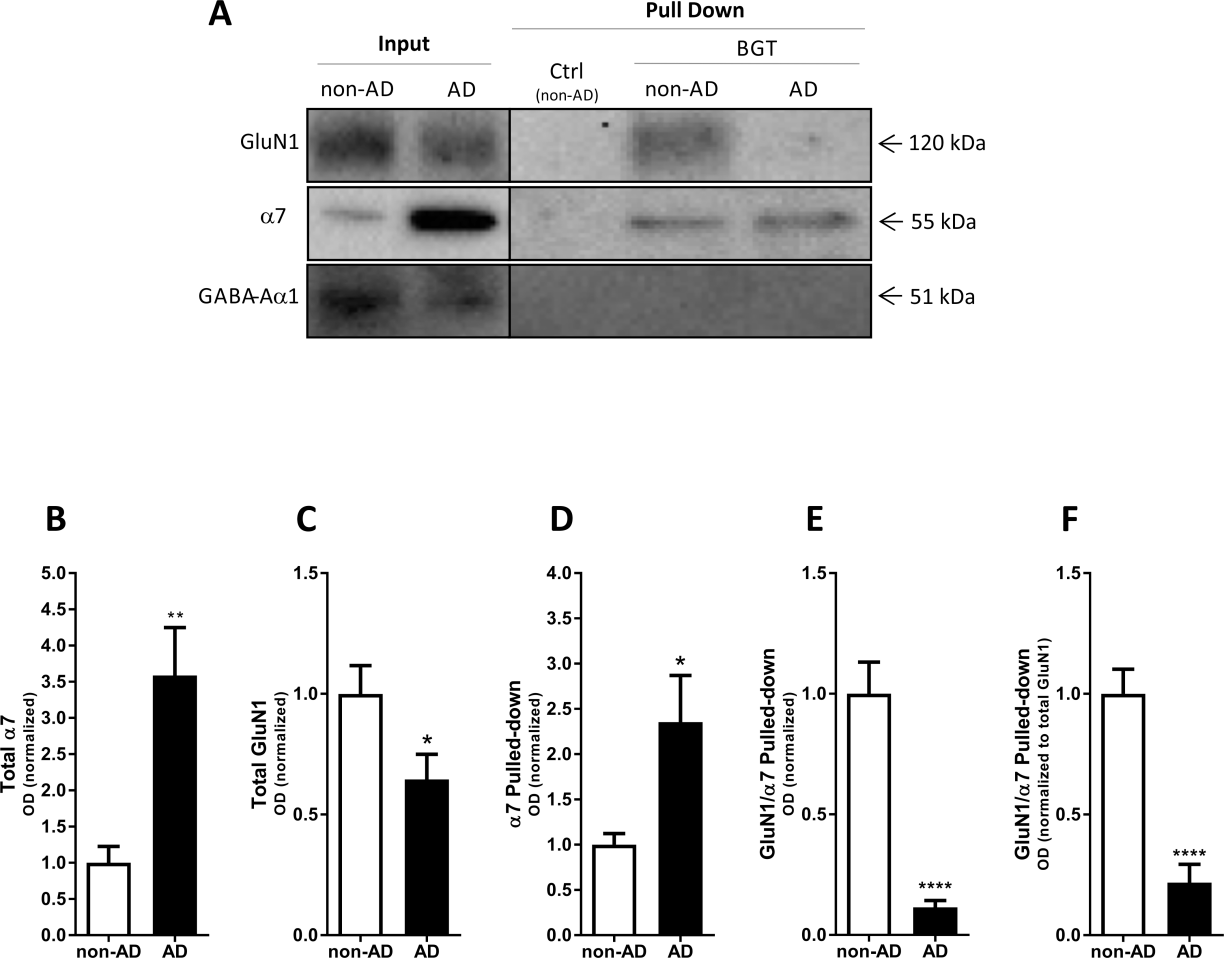
Complex formation between α7 nAChR and NMDAR in the AD brain. Affinity purification performed with agarose beads covalently coupled with α-bungarotoxin (BGT) or BSA (Ctrl) using homogenized postmortem surgically removed tissue of medial frontal gyrus from control subjects (non-AD) and from AD patients. (**A**) A representative example of a western blot illustrating GluN1, α7 nAChR and GABA_A_R α1 protein levels in total tissue lysates (Input) and pulled-down (Pull Down) samples from non-AD and AD cortical homogenates. (**B-C**) Quantification of total GluN1 (B) and total α7 (C) in lysates from non-AD and AD homogenates (both normalized to stain-free gel). (**D-F**) Quantification of α7 pulled-down (normalized to stain-free gel) **(D)**, of GluN1 pulled-down with α7 (normalized to the pulled-down α7) (E), and of GluN1 pulled-down with α7 (data normalized to pulled-down α7 and then further normalized to total GluN1 levels in input samples) (F). In B-F, the control group (non-AD) is set to 1, and values are shown as mean ± SEM. *p < 0.05, **p < 0.01 and ****p < 0.0001 indicate statistical significant difference from control subjects in unpaired *t*-tests, n = 8 (non-AD) and n = 7 (AD).

The post mortem brain tissues used for the experiments depicted in Fig 2 were from medial frontal gyrus of 7 Alzheimer Disease (AD) subjects and 8 control (non-AD) subjects (Table 1). These tissues were obtained from the Netherlands Brain Bank (Amsterdam, The Netherlands) and were the same as those used in a previous study [14]. Autopsies were performed on donors from whom written informed consent had been obtained either from the donor or direct next of kin. All AD subjects were confirmed by standard clinical [15] and neuropathologic [15, 16] diagnosis criteria.

**Table 1:** Clinicopathologic data of patients and human brain materials.

Diagnosis	Age	Gender	pH	PMD (h:min)	Braak stage
Non-AD	60	F	6.27	06:50	1
Non-AD	60	F	6.80	07:30	1
Non-AD	62	M	6.36	07:20	1
Non-AD	78	M	6.52	<17:40	1
Non-AD	87	M	7.11	08:00	1
Non-AD	87	F	6.91	08:00	2
Non-AD	97	F	—	10:00	2
Non-AD	90	F	6.54	06:10	3
AD	67	F	6.73	03:30	5
AD	58	M	6.29	05:15	6
AD	58	M	6.42	06:25	6
AD	59	M	6.26	05:05	6
AD	62	M	6.31	04:15	6
AD	62	F	6.53	04:25	6
AD	62	F	6.06	04:45	6

Keys: F, female; M, male; PMD, postmortem delay.

### Murine brain tissue

**Fig 3:**
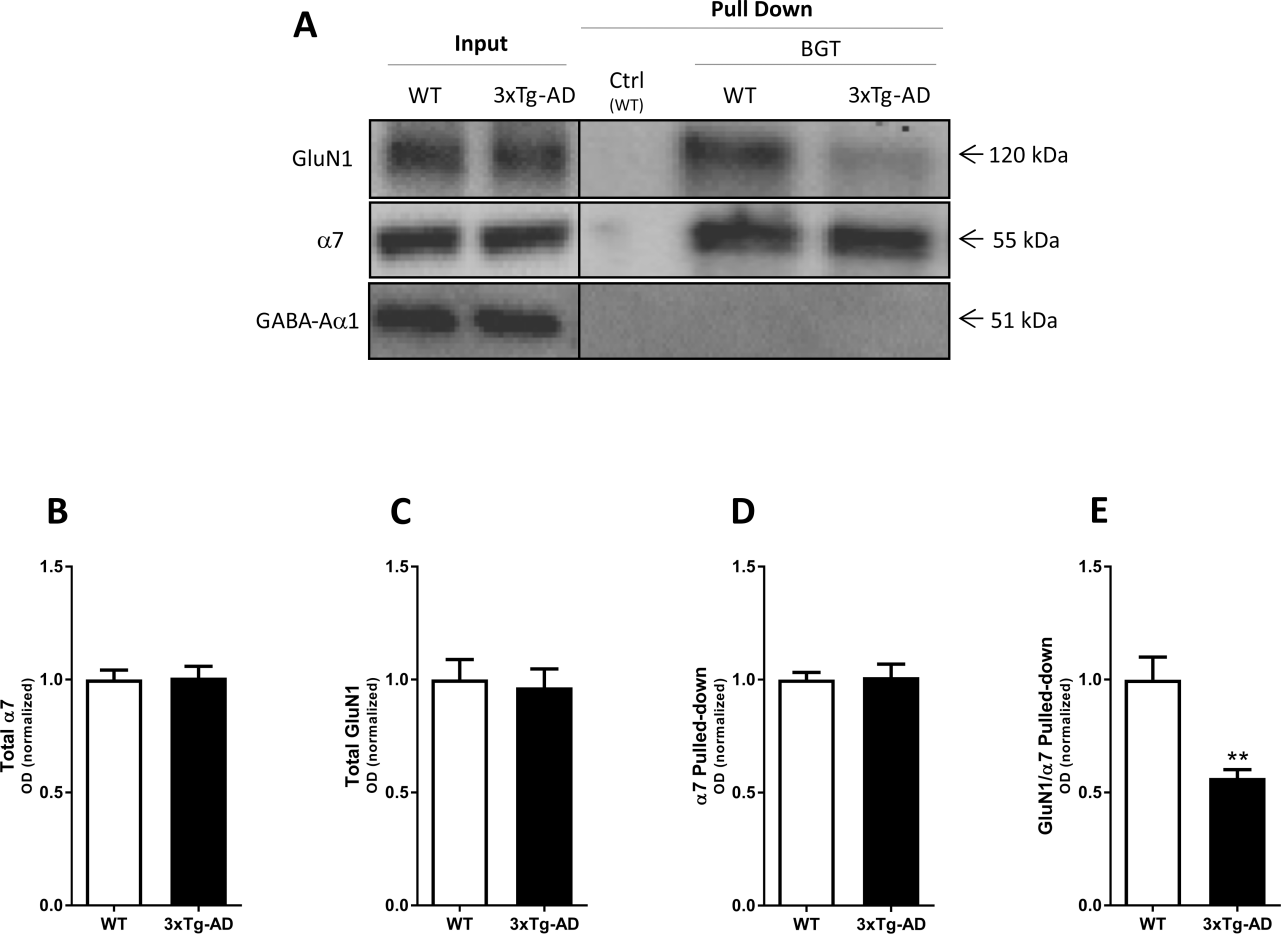
Complex formation between α7 nAChR and NMDAR in the adult 3xTg-AD mouse brain. Affinity purification performed with agarose beads covalently coupled with α-bungarotoxin (BGT) or BSA (Ctrl) using frontal cortical tissue lysates from adult 3xTg-AD mice (76–84 weeks old) and age- and sex-matched WT mice. (**A**) A representative example of a western blot illustrating GluN1, α7 nAChR and GABA_A_R α1 protein levels in total lysates (Input) and pulled down (Pull Down) samples from WT and 3xTg-AD mouse cortical homogenates. **(B-C)** Quantification of total GluN1 (B) and total α7 (C) in lysates from WT and 3xTg-AD mouse cortical homogenates (both normalized to stain-free gel). **(D-E)** Quantification of α7 pulled-down (normalized to stain-free gel) (D), and of GluN1 pulled-down with α7 (normalized to the pulled-down α7) (E). In B-E, the control group (WT) is set to 1, and values are shown as mean ± SEM. **p < 0.01 indicates statistical significant difference from WT group in unpaired *t*-tests, n = 8 (WT) and n = 8 (3xTg-AD).

**Fig 4:**
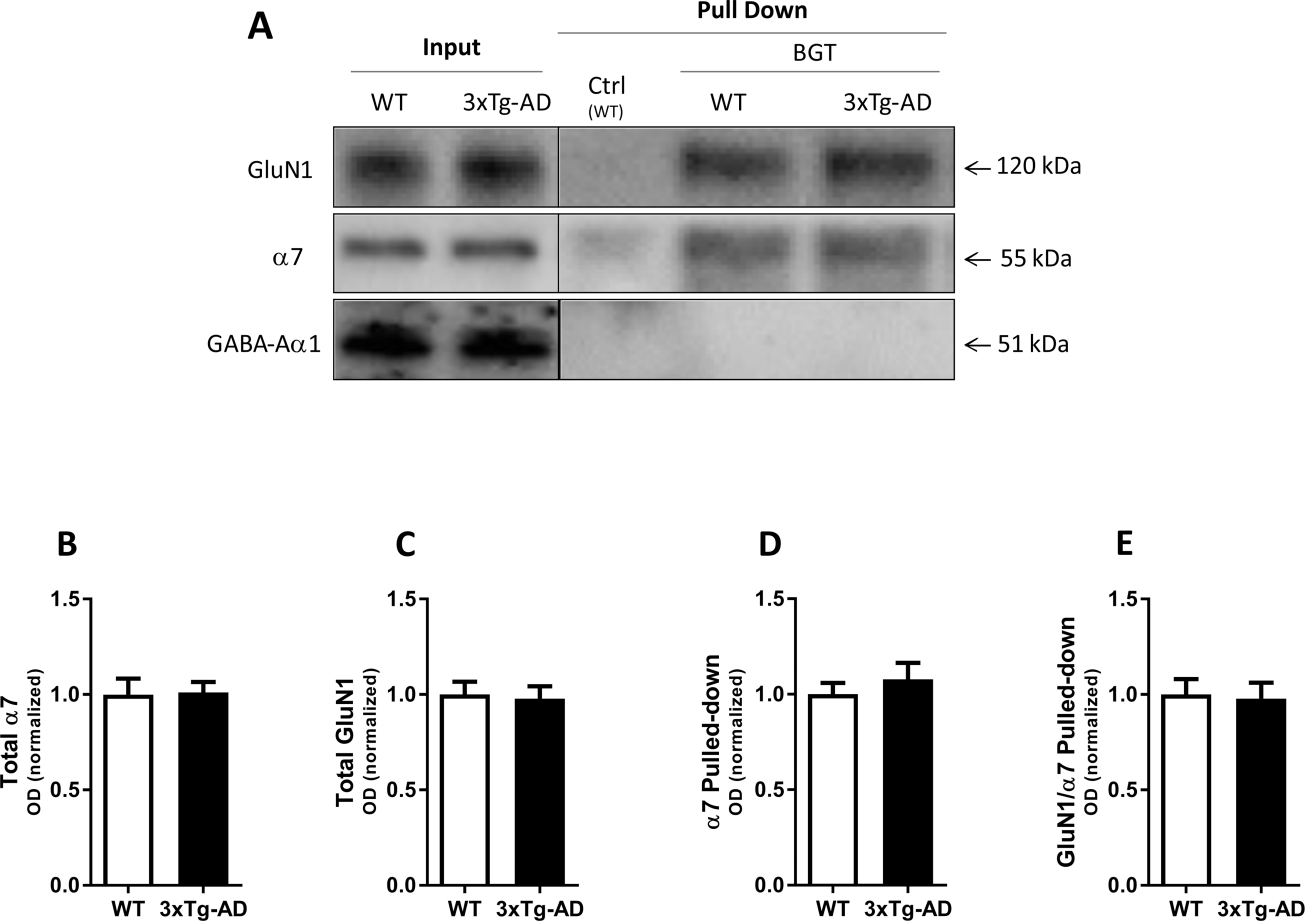
Complex formation between α7 nAChR and NMDAR in the young 3xTg-AD mouse brain. Affinity purification performed with agarose beads covalently coupled with α-bungarotoxin (BGT) or BSA (Ctrl) using frontal cortical tissue lysates from young 3xTg-AD mice (22–24 weeks old) and age- and sex-matched WT mice. (**A**) A representative example of a western blot illustrating GluN1, α7 nAChR and GABA_A_R α1 protein levels in total lysates (Input) and pulled down (Pull Down) samples from WT and 3xTg-AD mouse cortical homogenates. **(B-C)** Quantification of total GluN1 (B) and total α7 (C) in lysates from WT and 3xTg-AD mouse cortical homogenates (both normalized to stain-free gel). **(D-E)** Quantification of α7 pulled-down (normalized to stain-free gel) (D), and of GluN1 pulled-down with α7 (normalized to the pulled-down α7) (E). In B-E, the control group (WT) is set to 1, and values are shown as mean ± SEM, and statistical analysis for differences from WT group in unpaired *t*-tests was performed, n = 7 (WT) and n = 8 (3xTg-AD).

The cortical and hippocampal tissues from NMRI mice (2 females aged 14 weeks old and 2 males aged 10 weeks old) were used for the experiments depicted in Fig 1A and 1D. α7 nAChR knockout (KO) mice and WT littermates (C57BL/6 background) were purchased from The Jackson Laboratories and bred at Virginia Commonwealth University (Dr. Imad Damaj), and two α7 KO mice (1 male aged 8 weeks and 1 female aged 12 weeks) and two age- and sex-matched WT littermates (controls) were used for the experiments depicted in Fig 1C. Frontal cortices from adult (76–84 weeks old) 3×Tg-AD mice (n = 8) and age- and sex-matched WT controls (n = 8) were dissected and used for experiments depicted in Fig 3, and frontal cortices from young (22–24 weeks old) 3×Tg-AD mice (n = 8) and age- and sex-matched WT littermates (controls) (n = 7) were dissected and used for experiments depicted in Fig 4 [17]. All mice were sacrificed by decapitation, and their brains were separated and stored at -80°C. The manager of the vivarium had all the necessary licenses for housing laboratory animals.

### Preparation of soluble oligomeric Aβ_1–42_

Freshly made Aβ_1–42_ oligomers were prepared according to previously described methods [18, 19]. Briefly, Aβ_1–42_ was dissolved at a concentration of 1 mM in 1,1,1,3,3,3-hexafluoro-2-propanol and aliquoted. The volatile solvent was allowed to evaporate at RT (for about 2 h) until no visible liquid was left in the tube, leaving a film of Aβ_1–42_ in the tubes that were stored at -80°C. Immediately prior to use, the film was re-suspended in anhydrous DMSO (to a concentration of 5 mM) by 20 sec vortexing at RT, and diluted to the desired final concentration in phosphate-buffered saline (PBS). The solutions were vortexed, sonicated for 2 min, incubated for 24 h at 4°C, and immediately used for the experiment. The sizes of the Aβ_1–42_ oligomers formed were investigated by native western blotting and by transmission electron microscopy and the preparation was found to contain Aβ_1–42_ 10- and 16-mers (S1 Fig).

### Membrane protein fractionation and pull-down assay

Membrane protein lysates were prepared from human and mice tissues based on protocols adapted from previous studies [20, 21]. The tissues were homogenized in 1.5 ml (cortex) or 1 ml (hippocampus) of lysis buffer (50 mM Tris, 50 mM NaCl, 5 mM EDTA, 5 mM EGTA, 3 μl/ml protease inhibitor cocktail (Sigma), pH 7.5) using a PT1200C polytron blender (Kinematica, Luzern, Switzerland) for 4 × 5 sec. The lysates were centrifuged in an Optima™ LE-80K ultracentrifuge (Beckman Coulter) at 36.000 rpm at 4°C for 40 min, after which the supernatant was discarded. The pellets were then resuspended in the same volume of lysis buffer containing 2% Triton X-100, homogenized again for 4 × 5 sec and incubated for 2 h at 4°C on a rotor (15 rpm). Then the samples were centrifuged at 36.000 rpm at 4°C for 40 min, and the resulting supernatants were used for affinity purification (the pull-down assay). Total protein concentrations were determined colorimetrically using the Pierce 660 nm Protein Assay Reagent (Thermo Scientific, Rockford, IL).

The affinity purification using the pull-down method is based on the use of BGT-coated beads, with BSA-coated beads being used as negative control. In brief, BGT or BSA was dissolved in coupling buffer (0.1 M NaHCO_3_ buffer containing 0.5 M NaCl, pH 8.5) at 2 mg/ml. Next, the bait proteins were coupled to pre-washed N-hydroxysuccinimide (NHS)-activated agarose beads slurry in Pierce™ centrifuge columns (both Thermo Scientific) in a 1:1 (v/v) ratio. The bait proteins were then incubated with the beads for 2 h at RT while rotating. Successful coupling was confirmed by subsequent determination of BGT and BSA in the flow through. In those cases where coupling efficacy for BGT or BSA was found to be lower than ~85%, the coupling step was repeated until this coupling efficacy was achieved. Subsequently, the coupled beads were suspended in coupling buffer supplemented with 1 M ethanolamine for 30 min at RT while rotating in order to block any unreacted NHS-amino groups. Quenched coupled beads were then subjected to three washing rounds consisting of alternating washes with 1 ml coupling buffer and 1 ml 0.1 M acetate buffer (pH 4). After a final wash with coupling buffer, the beads were washed twice with lysis buffer.

From each sample, 100 μl beads (50% suspended in lysis buffer) were incubated with 1000 μg total protein in a total volume of 1500 μl lysis buffer for 17–20 h at 4°C on a rotor (15 rpm). In the experiments with the α7-pep2/α7-pep1 and Aβ_1–42_ peptides, the tissue lysates were preincubated with buffer or buffer supplemented with various concentrations of the respective peptides for 20 min at RT (α7-pep2 and α7-pep1) or for 1 h on ice (Aβ_1–42_) on a rotor (15 rpm) prior to this 17–20 h incubation. The time periods and temperatures used for these preincubations were based on those used in previous studies with α7-pep2/α7-pep1 and Aβ_1–42_ [11, 21]. The following day, the beads in the columns were washed with 3 x 500 μl phosphate buffer A (1 M NaCl, 8 mM Na_2_HPO_4_, 2 mM NaH2PO_4_, 0.5% Triton X-100, pH 7.5), and then with 3 x 500 μl phosphate buffer B (0.1 M NaCl, 8 mM Na_2_HPO_4_, 2 mM NaH_2_PO_4_, 0.5% Triton X-100, pH 7.5) and vortexed for 10 sec in between these washes. The beads in columns were collected in PBS, transferred into tubes and immediately processed for western blotting.

### Western blotting

Diluted protein samples and pulled-down agarose beads were boiled with loading buffer (in final concentrations of 60 mM Tris, 10% (v/v) glycerol, 2% (w/v) SDS, 5% (v/v) mercaptoethanol, 0.025% (w/v) bromophenol blue, pH 6.8) at 96°C for 10 min, and then allowed to cool on ice for 5 min. Equal amounts of protein (6 μg/lane) were loaded onto gel electrophoresis using Any kD^TM^ precast polyacrylamide gels (Biorad, Hercules, CA). Proteins on the gel were subsequently transferred to midi-size polyvinylidene fluoride (PVDF) membrane (Bio-Rad, Hercules, CA) with transfer buffer using Trans-Blot® TurboTM Transfer system (Bio-Rad, Hercules, CA) with the setting of 2.5A and 25V for 35 min. Membranes were then washed for 4 x 5 min with Tris-buffered saline, TBS (20 mM Tris and 150 mM NaCl, pH 7.5), supplemented with 0.05% (v/v) Tween-20 (TBS-T) and blocked in TBS supplemented with 5% (w/v) dry skim milk (Biorad) while gently shaking for 1 h at RT. Next the membranes were incubated in blocking buffer supplemented with primary antibodies against α7 (1:1000, #M220, Sigma), γ-aminobutyric acid type A receptor (GABA_A_R) α1 subunit (1:500, sc-7348, Santa Cruz Biotechnology, Heidelberg, Germany), β-actin (1:2000, #A5060, Sigma) or NMDAR subunits GluN1 (1:500, #556308) or GluN2A (1:250, #612286) (BD Pharmingen, New Jersey, USA) for 14–16 h at 4°C in a humidified chamber. Then the membranes were washed for 4 × 5 min in TBS-T and incubated for 1 h at 23°C in the blocking buffer supplemented with the corresponding horse radish peroxidase-conjugated secondary antibodies (Dako, Glostrup, Denmark). After washing the membranes in TBS-T for 4 × 5 min, protein bands were detected by enhanced chemiluminescence Western Lightning ECL Pro (Perkin Elmer, Waltham, MA) and visualized using Molecular Imager^®^ ChemiDoc^TM^ XRS+ imaging system (Biorad) with its ImageLab program for quantification of blots. Means of bands optical densities were measured and their corresponding background subtracted.

The primary antibodies used for the GluN1, GluN2A, GluN2B and GABA_A_R α1 subunits in the western blotting experiments have been shown to be specific for their respective proteins. The specificity of the GluN1 antibody has been verified on WT and GluN1 KO mice (region-specific deletion of the gene in cortex) by western blotting [22]. The specificity of the GluN2A antibody has been verified on forebrain tissue from WT and mutant mice deficient in GluR_epsilon-1_ (GluN2A) in western blotting [23], and the GluN2A and GluN2B antibodies have been shown to be specific in western blotting studies on tissue from GluN2A^(CTR)^ and GluN2B^(CTR)^ mutant mice [24]. The GABA_A_R α1 antibody has been used in western blotting studies demonstrating GABA_A_R α1 expression in rat brain, cerebellum and retinal tissues and using rat heart tissue as a negative control [25, 26]. The specificity of the α7 nAChR antibody will be addressed in detail in the *Results* section.

### Statistical analyses

All data in this study are presented as mean ± S.E.M. values. The statistical analysis was performed using GraphPad Prism version 7 for Windows (GraphPad Software, San Diego, CA). Data from the affinity purification experiments analyzed with Kruskal-Wallis test followed by Dunn’s multiple comparison test (multiple groups) or unpaired student’s *t*-test (two groups). *p* < 0.05 was considered statistically significant.

## Results

### Complex formation between α7 nAChR and NMDAR in murine and human brain tissue

As outlined in the *Introduction*, previous studies by the Liu group have demonstrated complex formation between native α7 nAChRs and NMDAR in rodent brain tissue by means of co-immunoprecipitation techniques [11–13]. In the present study, we initially set out to challenge these observations and investigated the putative formation of α7 nAChR/NMDAR complexes in both murine and human brain tissues. For this, we used another experimental set-up than that used by the Liu group, performing affinity purification using bead-coupled BGT. In this method, the highly selective high-affinity α7 nAChR antagonist BGT serves as an alternative approach to antibodies to affinity purify α7 nAChRs and the proteins interacting with this receptor (the “Pull Down” sample) from the total protein in the homogenates (the “Input” sample).

As can be seen from Fig 1A, the GluN1 subunit was co-purified with α7 pulled-down from both mouse cortical and hippocampal homogenates, as evidenced by the bands detected by antibodies for the GluN1 and α7 subunits with approximate molecular masses of 120 kDa and 55 kDa, respectively. In parallel, we also found GluN1 to be co-purified with α7 pulled-down from human cortical and hippocampal homogenates (Fig 1B). Importantly, the GABA_A_R α1 subunit was not detected in significant levels in the purified samples, even though the subunit in agreement with the literature clearly was expressed in both cortical and hippocampal lysates (Fig 1). Moreover, neither α7 nor GluN1 was detected in the pulled-down fractions obtained using beads coated with BSA for the affinity purification (Ctrl, Fig 1). We also found the GluN2A to be co-purified with α7 in the Pull Down samples (Fig 1D and 1E), but we consistently obtained more robust signals using the antibody for GluN1 than that for GluN2A. Since GluN1 is an integral subunit in all NMDARs *in vivo* [5] and detection of this subunit in the Pull Down samples thus is representative for the receptors, we decided to focus on this subunit throughout the study.

To further validate the apparent α7 nAChR/NMDAR complex formation observed in these experiments, we next studied the co-purification of the two receptors from cortical tissue from α7 KO mice [20]. Whereas both α7 and GluN1 was detected in the pulled-down sample from WT mouse cortex homogenate (analogously to the data in Fig 1A), neither of the two subunits could be detected in the corresponding sample from α7 KO cortical homogenate (Fig 1C). Another important observation made in this control experiment was the complete lack of α7 subunit in BGT pull-down from α7 KO homogenate, which contrasted the substantial band observed in the “Input” lane loaded with α7 KO homogenate (Fig 1C, S2 Fig). Several commercial α7 antibodies have been shown to be non-selective [27, 28], and this property also seems to apply for the antibody used in this study. However, considering the complete lack of an “α7-sized” band in the BGT pull-down from α7 KO homogenate, the α7-sized bands detected in the BGT pull-downs from homogenates from WT murine tissue (and human tissues) reflect specific immunodetection of the α7 nAChR with negligible non-specific binding of the antibody to other proteins. Hence, the use of the specific α7 nAChR antagonist BGT for the pull down means that none of the “non-α7” proteins targeted by this α7 antibody is present in the Pull Down sample, and the subsequent immunodetection of α7 using the antibody is thus a true reflection of the α7 protein present in the Pull Down sample.

In the next experiments, we probed the sensitivity of the apparent α7 nAChR/NMDAR complex formation in murine cortical homogenate to the α7-pep2 and α7-pep1 peptides. α7-pep2 (a 10-residue peptide with a sequence identical to the Leu^336^-Met^345^ region of the second intracellular loop of α7) has been shown to eliminate the co-immunoprecipitation of GluN2A with α7 nAChR, while α7-pep1 (a 10-residue peptide with a region identical to the Arg^316^-Gly^325^ fragment of the second intracellular loop of α7) was shown to be without effect [11]. Preincubation with α7-pep2 (10 μM and 50 μM) or α7-pep1 (10 μM and 50 μM) did not affect the levels of α7 pulled-down from the homogenates substantially (Fig 1D and 1E). In contrast, preincubation with α7-pep2 resulted in significantly reduced levels of GluN1 in the pulled-down sample (56% and 74% reductions observed for 10 μM and 50 μM α7-pep2, respectively), whereas preincubation with α7-pep1 (10 μM and 50 μM) did not change GluN1 levels substantially compared to the control samples (Fig 1D and 1E). Analogously, the level of the GluN2A subunit in pulled-down samples was substantially reduced by preincubation with 10 μM α7-pep2 and significantly reduced by preincubation with 50 μM α7-pep2 (47% and 58% reductions, respectively) but not by preincubation with α7-pep1 (10 μM and 50 μM) (Fig 1D and 1E).

All in all, the co-purifications of α7 nAChR and NMDAR subunits (mostly GluN1) from murine and human cortices and hippocampi are supported by all findings in the concomitantly performed control experiments. The complete absence of the subunits in Pull Down samples in experiments using BSA-coated beads or α7 KO cortical homogenate strongly suggest that any protein detected in the pulled-down sample is attributable to the α7 nAChR/BGT interaction, and that the presence of GluN1 and GluN2A (but not GABA_A_R α1) in the samples thus likely can be ascribed to an interaction between α7 and the NMDAR, either directly or via other proteins. The ability of the small α7-pep2 peptide to antagonize this interaction further supports that the co-purification of the receptors arises from a specific interaction.

### Complex formation between α7 nAChRs and NMDARs in AD brain tissue

In view of the key roles of glutamatergic and cholinergic transmission in processes underlying cognitive functions and the dysfunctions in these neurotransmitter systems associated with AD and other forms of dementia, we decided to investigate the formation of the α7 nAChR/NMDAR complex in AD brain tissue. Applying the same method as in the experiments above, the levels of the α7 and GluN1 subunits in pull-down samples from homogenates from post mortem surgically removed tissue of medial frontal gyrus from 7 AD patients and from 8 individuals not suffering from AD (non-AD) were investigated.

The expression levels of both α7 and GluN1 in the homogenates from the non-AD and AD cortical tissues were found to differ significantly (see Input lanes in Fig 2A). While the apparent expression of α7 nAChR in the AD homogenate was dramatically increased (258%) compared to the non-AD homogenate, the expression level of GluN1 was significantly decreased (36%) in the AD homogenate compared to the non-AD homogenate (Fig 2B and 2C). The observation for GluN1 was in concordance with findings in several studies of NMDAR or GluN1 expression levels in cortical [29, 30] and hippocampal [31] autopsies from AD patients. While most studies of α7 nAChR expression in AD cerebral cortex tissues have found receptor levels to be moderately decreased (reviewed in [32, 33]), some studies have also observed increased α7 nAChR expression (see for example [34, 35]).

Not surprisingly, the substantially higher total α7 nAChR expression in the AD cortical lysates resulted in ~2-fold higher levels of α7 nAChR in the pull-downs from AD homogenates compared to non-AD homogenates (Fig 2D). Interestingly, the GluN1/α7 ratio detected in the purified samples from AD homogenate was dramatically lower than that from non-AD homogenate (Fig 2E). This difference also existed when the GluN1/α7 ratio pulled-down was normalized to total GluN1 in the homogenates, thus taking the lower GluN1 expression in the AD lysates compared to the non-AD control lysates into account (Fig 2F). Thus, co-purification of the GluN1 subunit with α7 was reduced with 89% (Fig 2E) and 79% (Fig 2F) in the AD samples compared to the non-AD. The reduced GluN1 levels in the pulled-down samples from AD compared to non-AD cortical homogenates are also directly visible from the representative data presented in Fig 2A.

### Complex formation between α7 nAChR and NMDAR in brain tissue from 3xTg-AD mice

The apparent decreased degree of α7 nAChR/NMDAR complex formation detected in the human AD cortical homogenate compared to its control group prompted us to investigate whether the same reduction could be observed in 3xTg-AD mice compared to WT mice. The 3xTg-AD mouse is a triple-transgenic mouse model for AD harbouring PS1-M146V, APP(Swe) and tau-P301L transgenes that exhibit an age-related neuropathological progression pattern and comprises both amyloidosis and tau pathology and develops plaques and tangles [17]. Aβ load and Aβ plaque numbers as well as tau hyperphosphorylation have been found to be dramatically increased in hippocampus and frontal cortex from 9–12 months olds old 3xTg-AD mice compared to 6 months old animals [36], and the mice also develop synaptic dysfunctions, including LTP deficits, in an age-dependent manner [17]. In view of these age-dependent differences in amyloidosis and tau pathology and the phenotypes of these mice, we investigated and compared the α7 nAChR/NMDAR complex formation in both adult and young 3xTg-AD mice to those in age-and sex-matched WT mice controls.

#### Adult 3xTg-AD mice

In contrast to the significant differences observed between total α7 and GluN1 expression in AD and non-AD cortical homogenates, the expression levels of the two subunits in cortical homogenates from adult 3xTg-AD mice (76–84 weeks old) and age-and sex-matched nontransgenic controls were highly comparable (Fig 3A–3C). The similar GluN1 levels observed in the cortical homogenates from the two mice is supported by a previous report finding no significant difference between GluN1 levels in hippocampal tissues from 3xTg-AD and WT mice [37]. As for α7 nAChR, expression levels of this receptor in different CNS regions of 3xTg-AD mice have previously been reported to be similar or modestly decreased compared to the levels in non-transgenic controls [38, 39]. The comparable expression levels of α7 and GluN1 in adult 3xTg-AD and adult WT mouse cortex allowed us to make a direct comparison of levels of the two subunits detected in the pulled-down samples from the two homogenates. As one would expect from BGT-based affinity purification from two homogenates characterized by comparable α7 nAChR expression, α7 levels detected in the pull-downs from 3xTg-AD and WT homogenates were very similar (Fig 3D). In contrast, the GluN1/α7 ratio determined in the 3xTg-AD pulled-down samples was significantly reduced (44%) compared to that determined in the control samples (Fig 3E). Thus, the absolute reduction in GluN1/α7 ratio in the 3xTg-AD pull-down compared to the WT control was somewhat lower than that observed in the analogous experiments using human AD and non-AD cortical homogenates (Fig 2E and 2F). Importantly, however, the fact that significantly reduced fractions of GluN1 was co-purified with α7 from both human AD and 3xTg-AD cortical homogenates strongly suggested that this could be a trait arising from AD-related processes.

#### Young 3xTg-AD mice

As outlined above, the expression levels of both α7 and GluN1 in the cortical homogenates from adult 3xTg-AD mice and their controls were comparable (Fig 3A–3C). Analogously, the expression levels of neither α7 nor GluN1 differed significantly in cortical homogenates from the young animals (22–24 weeks old) compared to age-and sex-matched nontransgenic controls (Fig 4A–4C). In the case of α7, this was also reflected in the comparable levels of α7 detected in the pull-down samples from the two mice (Fig 4D). Interestingly, in contrast to the different levels of GluN1 pulled-down with α7 from adult 3xTg-AD and adult WT mouse cortical homogenates, the GluN1/α7 ratios determined in the BGT pull-down samples from cortical homogenates from young 3xTg-AD and young WT mice were highly similar (Fig 4E). Thus, whether the levels of GluN1 detected in BGT pull-down samples from cortical homogenates from this triple-transgenic mouse AD model differed significantly compared to those in pull down samples from the WT controls was clearly dependent on the age of the 3xTg-AD mice.

### Complex formation between α7 nAChR and NMDAR is disrupted by oligomeric Aβ_1–42_

**Fig 5:**
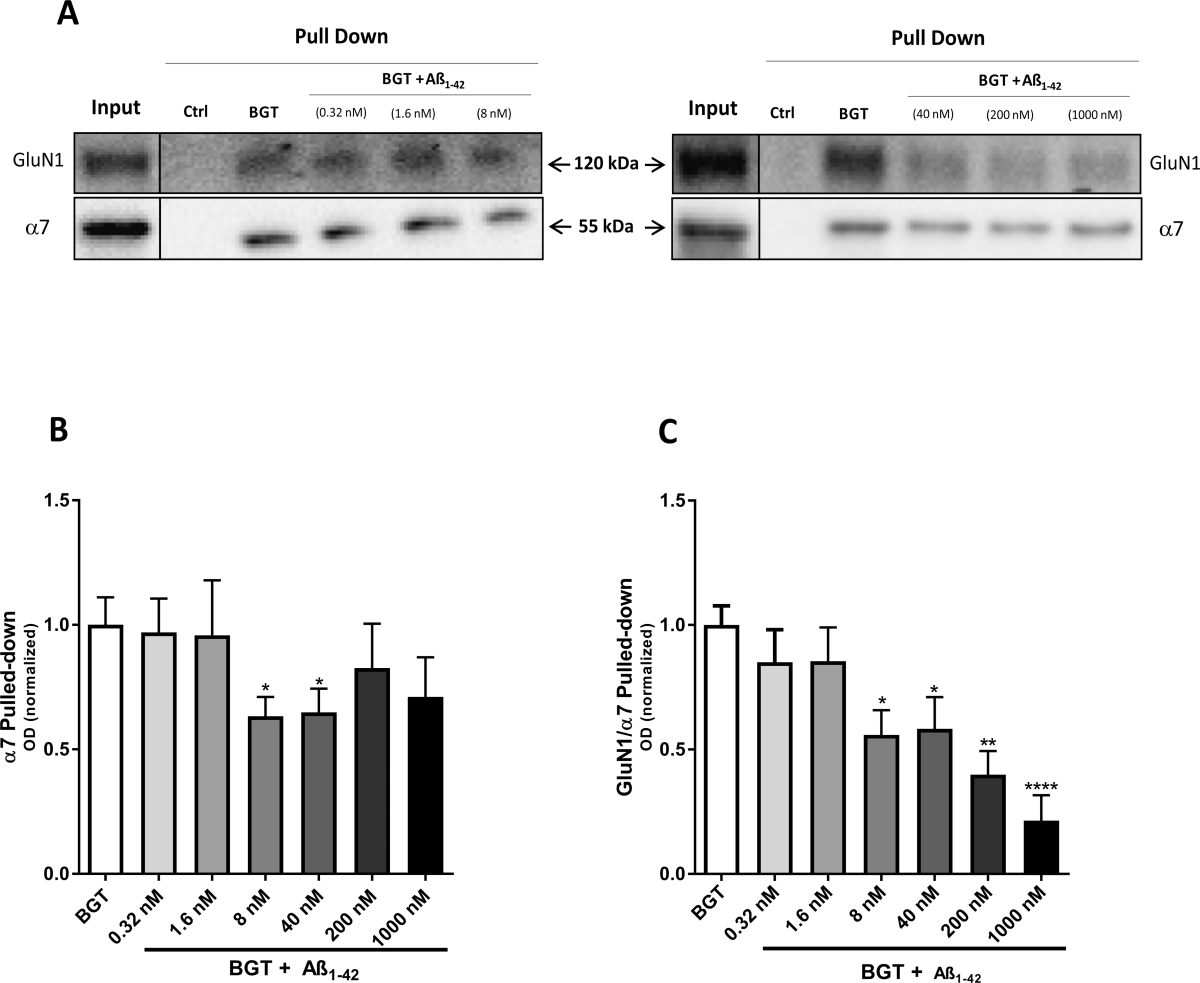
Complex formation between α7 nAChR and NMDAR in the presence of oligomeric Aβ_1–42_. Affinity purification performed with agarose beads covalently coupled with α-bungarotoxin (BGT) or BSA (Ctrl) using homogenates of human cortical tissue pretreated with various concentrations of oligomeric Aβ_1–42_. (**A**) A representative example of western blots illustrating GluN1 and α7 nAChR protein levels in total tissue lysates (Input) and pulled-down (Pull Down) samples from human cortical homogenates pretreated with buffer or buffer supplemented with various concentrations of oligomeric Aβ_1–42_. The two blots are from experiments performed in different days. (**B-C**) Quantification of α7 pulled-down (normalized to stain-free gel) (B) and GluN1 pulled-down with α7 (normalized to the pulled-down α7) (C). In B and C, the control group (BGT pull-down without Aβ_1–42_, given as “0” in the figure) is set to 1, and values are shown as mean ± SEM (n = 3). The experiments were performed in duplicate for **A**β_**1–42**_ concentrations 0.32, 1.6 and 8.0 nM and in triplicate for **A**β_**1–42**_ concentrations 40, 200 and 1000 nM using homogenates from three different human cortices. *p <0.05, **p < 0.01 and ****p < 0.0001 indicate statistical difference from the vehicle-treated group in a Kruskal-Wallis test with Dunn’s multiple comparison test.

Next, we sought to elucidate the molecular mechanism underlying the significantly reduced degrees of co-purification of α7 nAChR and NMDAR from the cortical homogenates from human AD compared to non-AD controls and from adult 3xTg-AD mice compared to age-matched controls. Here our attention turned to the Aβ_1–42_ peptide, a main component of the amyloid plaques formed in AD and one of the key pathological hallmarks of the disease [40, 41], which also has been found to be expressed at substantially higher levels in adult 3xTg-AD mouse brain tissue than in young animals [17, 36]. This prompted us to investigate whether Aβ_1–42_ in its oligomeric form could affect the formation of the α7 nAChR/NMDAR complex in human cortical homogenates (from non-AD individuals) (Fig 5).

Whereas pretreatment of human cortical homogenates with low Aβ_1–42_ concentrations (0.32 and 1.6 nM) did not change the levels of α7 in the BGT pull-down samples, higher peptide concentrations (8.0, 40, 200 and 1000 nM) resulted in somewhat reduced levels of the subunit in the pulled-down samples, with 8 nM and 40 nM Aβ_1–42_ giving rise significant decreases of 37% and 35%, respectively (Fig 5B). More strikingly, whereas pretreatment with low Aβ_1–42_ concentrations (0.32 and 1.6 nM) did not affect the levels of GluN1 co-purified with α7 significantly, higher Aβ_1–42_ concentrations induced robust reductions in the GluN1/α7 ratio in the pulled-down samples, with 8, 40, 200 and 1000 nM Aβ_1–42_ giving rise to 44%, 42%, 60% and 79% reduced ratios compared to the control (Fig 5C). Importantly, it should be stressed that the reduced α7 levels detected in the BGT pull-down samples from homogenate pretreated with the higher Aβ_1–42_ concentrations (Fig 5B) can not explain the reduced GluN1/α7 ratios in the same samples: if anything, the reduced α7 levels in these samples could mean that the reductions in the GluN1/α7 ratio have been underestimated. In conclusion, pretreatment with oligomeric Aβ_1–42_ seemed to antagonize the formation of the α7 nAChR/NMDAR complex in the human cortical homogenate in a concentration-dependent manner.

## Discussion

The fairly recent realization that the trafficking, cellular distribution and function of neurotransmitter receptors can be dynamically modulated by their assembly into multimeric complexes has introduced another level of complexity to neurotransmission, and particularly the functional implications of GPCR oligomerization have been explored extensively [42]. The trafficking and signaling of LGICs are regulated via their interactions with various intracellular scaffold and adaptor proteins [43–45], and the receptors have also been proposed to form complexes with GPCRs [46–50]. While the proposed α7 nAChR/NMDAR complex was the first report of complex formation between two LGICs, the recent demonstration of direct interaction and cross-inhibition between α6-containing nAChRs and P2X2/3 purinergic receptors in dorsal root ganglia cells [51] further supports the notion that members from different LGIC families can assemble into multimeric complexes.

To date, the α7 nAChR/NMDAR complex formation proposed by the Liu group [11–13] has not been verified by other groups. This work thus represents the first data from another lab supporting the existence of this complex in brain tissues, and it also provides additional insights into the assembly. Applying affinity purification based on BGT-coupled beads and subsequent immunodetection of α7 and NMDAR subunits in the BGT pull-down, the two receptors have been co-purified from murine and human brain tissues (Fig 1A and 1B). Together with the original demonstration of the complex in rat brain tissue [11], these data suggest that the complex may exist in several species, including the human brain. Moreover, whereas Li et al. co-immunoprecipitated α7 nAChR and NMDAR from rat hippocampal and amygdala tissues but not from prefrontal cortex homogenate [11], we consistently co-purified the receptors from hippocampal as well as cortical tissues, suggesting that this complex formation is widespread in the brain (Fig 1A and 1B). This apparent discrepancy between the findings in Liu et al. [11] and this work could be rooted in different sensitivities of the co-immunoprecipitation method and the affinity purification assay used here. Whereas BGT displays high affinity and selectivity for α7 nAChRs, commercial α7 antibodies have consistently been found to be non-selective [27, 28], and the use of BGT for the pull-down may thus provide a higher signal-to-noise ratio that could facilitate co-purification of the receptors from tissues from additional brain areas.

The control experiments performed using the affinity purification assay collectively substantiate that the co-purification of α7 nAChRs and NMDARs from the human and murine brain tissues is reflective of a specific interaction between the two receptors. The absence of both α7 and GluN1 in BGT pull-down samples from α7 KO mice cortical homogenate both confirms the specificity of BGT-coated beads and that the intensities of α7 bands detected in the pull-down samples are reliable reflections of the actual α7 nAChR levels (Fig 1C). Furthermore, it also demonstrates that presence of GluN1 in the BGT pull-down is completely dependent on α7 nAChR being present in the homogenate, and the absence of GABA_A_R α1 in the pull-downs further supports the specificity of the α7 nAChR-NMDAR interaction (Fig 1). Finally, the reduced NMDAR subunit levels detected in the BGT pull-downs upon pretreatment of cortical homogenates with the α7-pep2 peptide also substantiate that the co-purification is the result of a specific interaction (Fig 1D and 1E).

While the results of the control experiments thus support the formation of the α7 nAChR/NMDAR complex, it is important to keep a general concern associated with use of biochemical approaches to demonstrate protein-protein interactions in mind [52]: the fact that the concentrations of all proteins in the homogenates will be vastly increased compared to the endogenous expression levels in neurons and that assay conditions differ substantially from those in native tissues could potentially trigger assembly of proteins into complexes that are either not formed or are of a more transient nature *in vivo*. Moreover, the molecular basis for α7 nAChR/NMDAR assembly also remains to be fully elucidated. The complex has been proposed to be rooted in a direct interaction between α7 nAChR and GluN2A [11], but it seems premature to rule out the involvement of additional proteins (other than α7 and the NMDAR subunits) in the assembly. For example, α7 nAChRs associate with a wide range of scaffold and adaptor proteins, including PDZ-containing proteins such as PSD-95 that also interacts directly with the C-termini of NMDARs (reviewed in [53])

The significantly reduced degree of α7 nAChR/NMDAR complex formation detected in AD cortical homogenates compared to non-AD controls is perhaps the most interesting finding of this study (Fig 2). Importantly, the analogous reduction in GluN1 co-purified with α7 from cortical homogenates from adult 3xTg-AD mice compared to age-matched controls further supports that these reductions may be attributable to specific processes related to amyloidosis and/or tauopathy, in particular in the light of the comparable GluN1/α7 ratios observed in young 3xTg-AD and age-matched controls (Figs 3 and 4). As outlined in *Results*, the different GluN1/α7 ratios in the AD and non-AD pull-downs can not be explained by the different expression levels of α7 and GluN1 in the two tissues, and this is further evident from the different GluN1/α7 ratios in pull-down samples from adult 3xTg-AD and WT mouse cortical homogenates characterized by comparable expression levels of the two subunits (Figs 2 and 3). Instead, the reduced GluN1 levels detected in the BGT pull-downs from the human AD and adult 3xTg-AD homogenates compared to their respective controls appear to arise from an impaired interaction between α7 nAChR and NMDAR, be it a direct interaction or one mediated via other proteins.

The reduced degree of α7 nAChR/NMDAR complex formation in AD compared to non-AD brain tissue could arise from numerous factors. However, the key role of Aβ_1–42_ for AD pathology [40, 41] and the clear age-dependent nature of the observed reduction in GluN1/α7 ratios in the BGT pull-down samples from 3xTg-AD cortex (Figs 3 and 4) prompted us to investigate the effect of the peptide on the formation of this putative complex. The fact that oligomeric Aβ_1–42_ mediated reductions in GluN1 levels in the BGT pull-downs in a concentration-dependent manner offered a plausible molecular mechanism for the reduced α7 nAChR/NMDAR complex formation in the AD tissue (Fig 5). Whereas it has yet to be established conclusively whether Aβ oligomers bind NMDARs [54, 55], a substantial amount of evidence indicates that Aβ_1–42_ and other Aβ peptides act directly at α7 nAChRs (reviewed in [56]). However, the reported binding affinities and the modulatory potencies of Aβ_1–42_ at recombinant and native α7 nAChRs have varied considerably, which probably can be ascribed to the different assays, cell/neuron cultures and soluble oligomeric Aβ_1–42_ preparations used in the studies (reviewed in [56]). Thus, the nanomolar Aβ_1–42_ concentrations found to give rise to significantly reduced GluN1/α7 ratios in the BGT pull-downs in this study are in good agreement with some, but not all, of these previous findings (Fig 5C). Another pertinent question is whether the nanomolar concentrations of oligomeric Aβ_1–42_ antagonizing the α7 nAChR/NMDAR complex formation in human cortical homogenate can be said to be reconcilable with the reduced GluN1/α7 ratios observed in cortical tissues from the AD brain and adult 3xTg-AD mouse brain. i.e. whether or not the reduced ratios here can be ascribed to the endogenous Aβ_1–42_ concentrations in the diseased tissues (Figs 3–5). Such comparisons are tricky, since determinations of Aβ_1–42_ concentrations in AD brain tissues in the literature vary a lot depending on the analytical methods used, the brain tissue preparations investigated and other factors [57, 58], and since Aβ_1–42_ densities or concentrations not necessarily are accurate representations of the extracellular concentrations of the peptide. Aβ_1–42_ concentrations in cerebrospinal fluid (CSF) or brain interstitial fluid of AD patients have been reported to be in the 500–1000 pM range, and the concentrations of the peptide in 3xTg-AD mouse CSF have been determined to 0.5–1.5 μg/L (~100–300 pM) [41, 59]. Although these levels clearly are considerably lower than the effective concentrations exhibited by Aβ_1–42_ in the experiments in Fig 5C, it is not clear to which extent these CSF concentrations of Aβ_1–42_ are representative for the extracellular concentrations of the peptide in the AD brain. In light of this, we will refrain from drawing too rigid conclusions as to the molecular mechanism underlying the reduced α7 nAChR/NMDAR complex formation in AD brain tissue and the role of Aβ_1–42_ for it. It does seem plausible that binding of oligomeric Aβ_1–42_ to α7 could disrupt molecular interactions between the two receptors in the α7 nAChR/NMDAR complex, or between α7 and another protein in a multimeric complex, and break down the complex. On the other hand, the effect could also be rooted in a more indirect mechanism, since oligomeric Aβ_1–42_ and other Aβ peptide mediates a wide range of molecular and cellular effects in the AD brain [40, 41]. Finally, we cannot rule out that non-amyloidogenic processes in the AD brain could contribute significantly to the reduced α7 nAChR/NMDAR complex formation in AD brain tissue.

## Conclusion

The present work suggests that the α7 nAChR/NMDAR complex is formed in both the rodent and human CNS, and that formation of this complex is significantly decreased in the AD brain compared to the healthy brain, with oligomeric Aβ_1–42_ being a plausible key molecular determinant of this difference. However, presently very little is known about the importance of such a complex in the healthy and diseased brain. Furthermore, demonstration of protein-protein interactions in native tissues beyond any reasonable doubt is not trivial and requires a substantial amount of evidence obtained by different methodologies. Thus, the present findings should be considered a contribution to an ongoing exploration of this putative complex and its physiological roles, and it will be important to confirm or challenge the existence of the complex *in vivo* in future studies based on other approaches. The sheer possibility of the formation of a complex comprised of Glu and ACh receptors that may be disrupted by Aβ peptides should warrant and fuel such explorations.

## Supporting information

S1 Fig **A.** Aβ_1–42_ oligomer formation assessed by native western blotting. Silver stain of the following proteins (lanes): A) Aβ_1–42_ monomers, B) Aβ_1–42_ oligomers, C) fibrils, D) N-DMEM. There is considerable background staining, and the sensitivity of the Aβ_1–42_ antibody is not great. Nevertheless, clear bands at around 40 and 55 kDa can be observed in lane B (shown by black box), suggesting that the prepared Aβ_1–42_ mixture contains 10-16-mers. **B.** Aβ_1–42_ oligomer formation assessed at different times (after 3, 6, 9 and 24 h) using transmission electron microscopy (TEM) imaging. The experiments were performed as previously described [60, 61]. Briefly, 2 μl of the diluted samples (20 μM) were prepared by placing on a carbon-coated grid. The samples were stained with 1% uranylacetate and then placed on a clean paper for removing excess staining solution. The grids were thoroughly examined using TEM (JEOL 1010, Japan).
(PDF)

S2 Fig The identity of the band detected with the the α7 antibody in the BGT pull-down samples. Total homogenates (pre pull-down, Input) and pulled-down samples from two α7 WT and two α7 KO mouse cortical homogenates were submitted to gel electrophoresis and western blotting followed by detection using the α7 antibody. This gel has been allowed to be developed to full saturation (which is the reason for the intensely red colored bands in several of the lanes). Analogously to the data in Fig 1C, the α7 antibody can detect bands (at approximately 55 kDa) in the total lysates from both WT mouse and α7 KO mouse cortical homogenates, demonstrating the non-specificity of the antibody. As can be seen from the right side of the gel, however, the intense bands (at approximately 55 kDa) observed in the two “α7 WT” lanes for the BGT pull-down samples are contrasted by the negligible bands or complete absence of bands in the two “α7 KO” lanes. This support the conclusion made based on Fig 1C in the manuscript: that the protein detected by the α7 antibody in the BGT pull-down samples is indeed the α7 nAChR.
(PDF)
