# Supplementary figures and images for: Probing the putative α7 nAChR/NMDAR complex in human and murine cortex and hippocampus: Different degrees of complex formation in healthy and Alzheimer brain tissue
Source: PLoS One. 2017 Dec 20;12(12):e0189513. doi: 10.1371/journal.pone.0189513 (PMC5738045; doi:10.1371/journal.pone.0189513)

## S1 Fig

**A**

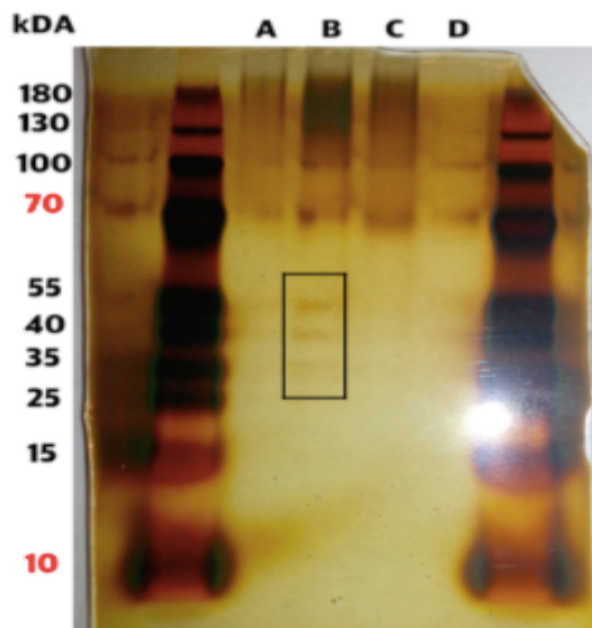

**B**

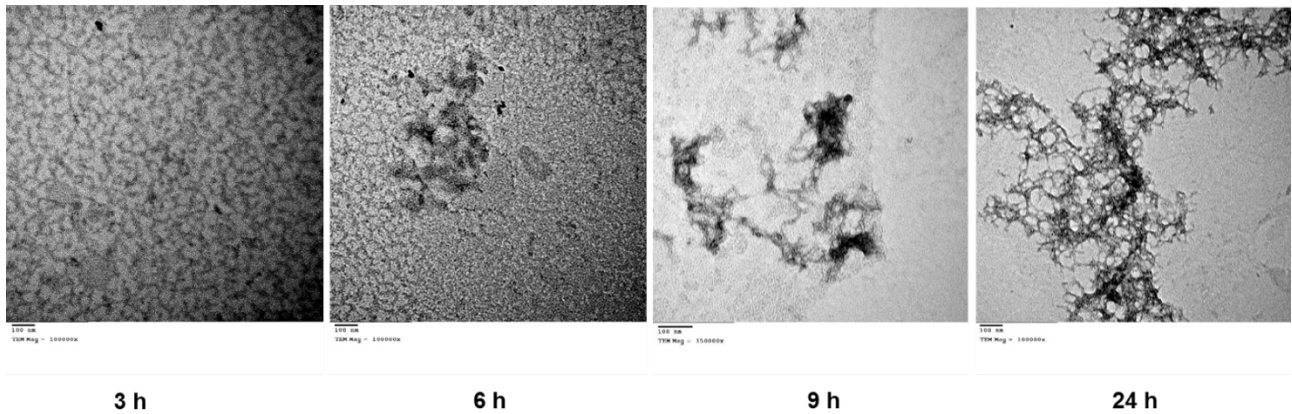

Supplement: S1 Fig — A. Aβ1–42 oligomer formation assessed by native western blotting. Silver stain of the following proteins (lanes): A) Aβ1–42 monomers, B) Aβ1–42 oligomers, C) fibrils, D) N-DMEM. There is considerable background staining, and the sensitivity of the Aβ1–42 antibody is not great. Nevertheless, clear bands at around 40 and 55 kDa can be observed in lane B (shown by black box), suggesting that the prepared Aβ1–42 mixture contains 10-16-mers. B. Aβ1–42 oligomer formation assessed at different times (after 3, 6, 9 and 24 h) using transmission electron microscopy (TEM) imaging. The experiments were performed as previously described [60, 61]. Briefly, 2 μl of the diluted samples (20 μM) were prepared by placing on a carbon-coated grid. The samples were stained with 1% uranylacetate and then placed on a clean paper for removing excess staining solution. The grids were thoroughly examined using TEM (JEOL 1010, Japan). (PDF) [file pone.0189513.s001.pdf]

## S2 Fig

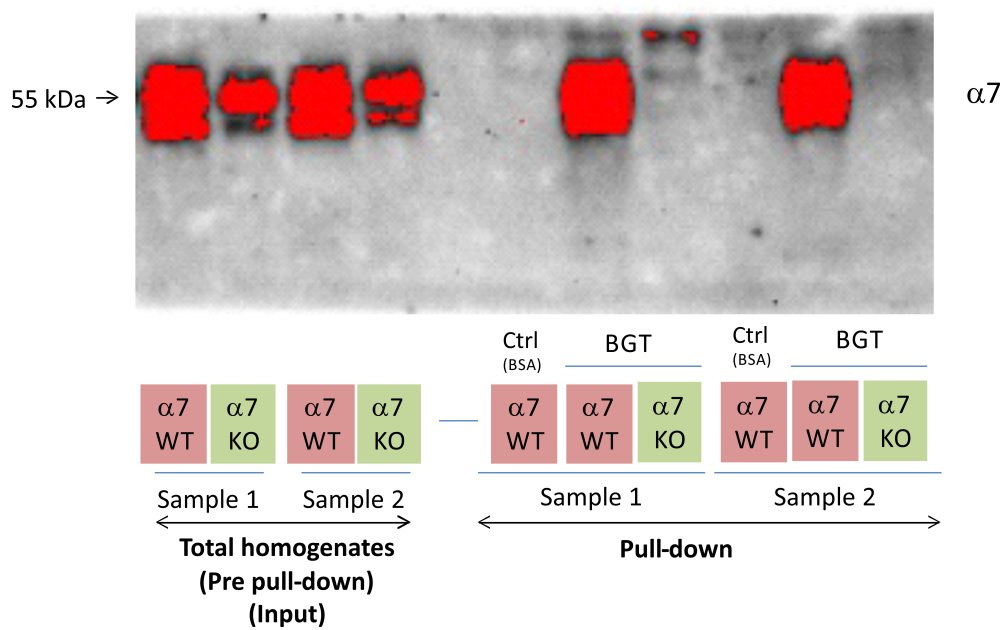

Supplement: S2 Fig — Total homogenates (pre pull-down, Input) and pulled-down samples from two α7 WT and two α7 KO mouse cortical homogenates were submitted to gel electrophoresis and western blotting followed by detection using the α7 antibody. This gel has been allowed to be developed to full saturation (which is the reason for the intensely red colored bands in several of the lanes). Analogously to the data in Fig 1C, the α7 antibody can detect bands (at approximately 55 kDa) in the total lysates from both WT mouse and α7 KO mouse cortical homogenates, demonstrating the non-specificity of the antibody. As can be seen from the right side of the gel, however, the intense bands (at approximately 55 kDa) observed in the two “α7 WT” lanes for the BGT pull-down samples are contrasted by the negligible bands or complete absence of bands in the two “α7 KO” lanes. This support the conclusion made based on Fig 1C in the manuscript: that the protein detected by the α7 antibody in the BGT pull-down samples is indeed the α7 nAChR. (PDF) [file pone.0189513.s002.pdf]
